# Role of microbial secreted proteins in gut microbiota-host interactions

**DOI:** 10.3389/fcimb.2022.964710

**Published:** 2022-07-29

**Authors:** Boris Vidal-Veuthey, Dámariz González, Juan P. Cárdenas

**Affiliations:** ^1^ Centro de Genómica y Bioinformática, Facultad de Ciencias, Ingeniería y Tecnología, Universidad Mayor, Huechuraba, Chile; ^2^ Escuela de Biotecnología, Facultad de Ciencias, Ingeniería y Tecnología, Universidad Mayor, Santiago, Chile

**Keywords:** secretome, gut microbiome, secretion systems, extracellular vesicles, probiotics, postbiotics

## Abstract

The mammalian gut microbiota comprises a variety of commensals including potential probiotics and pathobionts, influencing the host itself. Members of the microbiota can intervene with host physiology by several mechanisms, including the secretion of a relatively well-reported set of metabolic products. Another microbiota influence mechanism is the use of secreted proteins (i.e., the secretome), impacting both the host and other community members. While widely reported and studied in pathogens, this mechanism remains understood to a lesser extent in commensals, and this knowledge is increasing in recent years. In the following minireview, we assess the current literature covering different studies, concerning the functions of secretable proteins from members of the gut microbiota (including commensals, pathobionts, and probiotics). Their effect on host physiology and health, and how these effects can be harnessed by postbiotic products, are also discussed.

## Introduction

Human microbiota research has become a relevant subject in microbiology in the last few decades. Moreover, the relationships between microbiota, health, and disease have gained increasing attention, given their potential implications in human physiology and applications in human medicine (Cullen et al., 2020). The human microbiota contains an enormous genetic content; it includes an estimated set of 22 million protein-coding genes for the intestinal microbiota (Tierney et al., 2019), an overwhelming amount compared with the approximately 21,000 protein-coding genes found in the human genome (Pertea et al., 2018). This gene repertoire is involved in microbial adaptation to the human body and how those microbes interact with their niches across the human body. This enormous gene set from the human microbiota, known as the *human microbiome*, can induce changes in the host by the action of their gene products.

One of the most relevant mechanisms of this influence effect is releasing a variety of molecules into their niches (Shine and Crawford, 2021). From fermentation products to antimicrobial peptides, these molecules are involved in various processes influencing the host and other community members. For example, short-chain fatty acids (SCFAs) potentially affect the host metabolism and signaling, with implications for health and disease (Ratajczak et al., 2019; Dalile et al., 2019; Sanna et al., 2019). The study of the role of the SCFA on the relation between human microbiota and human cells is a relatively well-covered topic covered in other reviews (Tan et al., 2014; Zheng et al., 2022). Moreover, other reviews cover the effect and role of the pleiad of secreted metabolites on the interaction between human microbiota and the host and the interaction between microbial communities themselves (e.g., (Shine and Crawford, 2021)). Among the repertoire of secreted compounds produced by the microbiota, an increasingly relevant group of study targets corresponds to the poorly studied secretable proteins known as the secretome (Tjalsma et al., 2000).

Several findings concerning the structure and function of secretion systems and secreted proteins have been made formerly from studies using pathogenic microbes (Green and Mecsas, 2016; Nicod et al., 2017). In several cases, pathways connecting the secretion of different proteins with changes in the host cellular program are also proposed (Hueck, 1998; Bumann et al., 2002; van Ulsen and Tommassen, 2006), suggesting that secreted protein effectors result from a very specialized relationship between specific pathogens and their host. Likewise, there is a possibility that several commensal-associated proteins are involved in a particular relationship with the host. Therefore, the potential role of secreted proteins is gaining importance in understanding the human microbiome-host relationship dynamics. However, there is little evidence about the repertoire of secreted proteins produced by gut commensals. In the following minireview, we assess the current literature covering the main studies of secreted or exposed proteins as effectors on the host physiology in the gut microbiome. We also cover the known implications on host health and diseases and discuss future research projections in this field.

## Secretion mechanisms in the gut microbiota

In order to produce any effect on the host, secreted proteins require certain systems for their release. Some mechanisms involved in protein secretion among gut commensals and probiotic microbes are currently reported. For example, several gut commensals contain genes for different bacterial secretion systems, being protein complexes involved in the transport of proteins from the cytoplasm to the extracellular side of the cell (Green and Mecsas, 2016). Genes encoding components of the Type VI secretion system (T6SS) are widely distributed among Proteobacteria and Bacteroidetes (Silverman et al., 2012), and they were recovered from some members of the gut microbiota, such as *Bacteroides* (Russell et al., 2014), with even the presence of some genomic islands associated with those systems in strains of the common commensal *Bacteroides fragilis* (Coyne et al., 2016). The main roles of the effect proteins using these secretion systems are related to ecological competition between related microorganisms (Chen et al., 2019; Wood et al., 2020). T6SS may also have a modulatory role: a study on *Helicobacter hepaticus*, a pathobiont from the gut microbiota, showed that T6SS limited the ability of this microbe to colonize and induce inflammation, promoting a balanced relationship with the host (Chow and Mazmanian, 2010); the breakdown of this balancing role may have a role in certain diseases such as inflammatory bowel disease and colon cancer.

Another system for protein secretion in gut commensals, and some probiotic organisms, consists of extracellular vesicles (EVs) or outer membrane vesicles (OMVs). EVs are lipid-closed bodies produced by cells and released into the extracellular spaces (Doyle and Wang, 2019). Previous studies have shown that some gut commensals can produce EVs (Cortés et al., 2021; Díaz-Garrido et al., 2021; Ñahui Palomino et al., 2021). For example, a study analyzing EVs obtained in fecal samples from patients in different colorectal cancer (CRC) stages and healthy conditions (Park et al., 2021) could suggest the taxonomic origin for those EVs; vesicles from members of the Rikenellaceae and Acidaminococcaceae families were enriched in late CRC subjects, whereas Lactobacillaceae-derived EVs were relatively enriched in early CRC subjects. Another recent study (De Saedeleer et al., 2021), utilizing a methodological framework for the extraction of microbiome-derived, extracellular components, identified several extracellular proteins, mostly associated with Bacteroidetes and Proteobacteria, previously identified in OMVs; some of those proteins include ribosomal proteins, chaperones, outer membrane proteins, and some enzymes (glutamine synthetase, recombinase A, and formate acetyltransferase), as well as a variety of proteins involved in metabolite transport. Another study could isolate EVs from an enriched culture of porcine intestinal microbiota incubated with beta-mannan (Lagos et al., 2020), showing that several proteins enriched in those EVs were associated with translation, energy metabolism, and amino acid/carbohydrate transport. These studies confirm the occurrence and importance of EVs/OMVs in the intestinal niche from human and non-human hosts (Cecil et al., 2019) and the relevance of those vesicles as a powerful new approach for therapeutic approaches (Morishita et al., 2021).

## Secretable proteins produced by commensals, pathobionts, and probiotics

**Figure 1:**
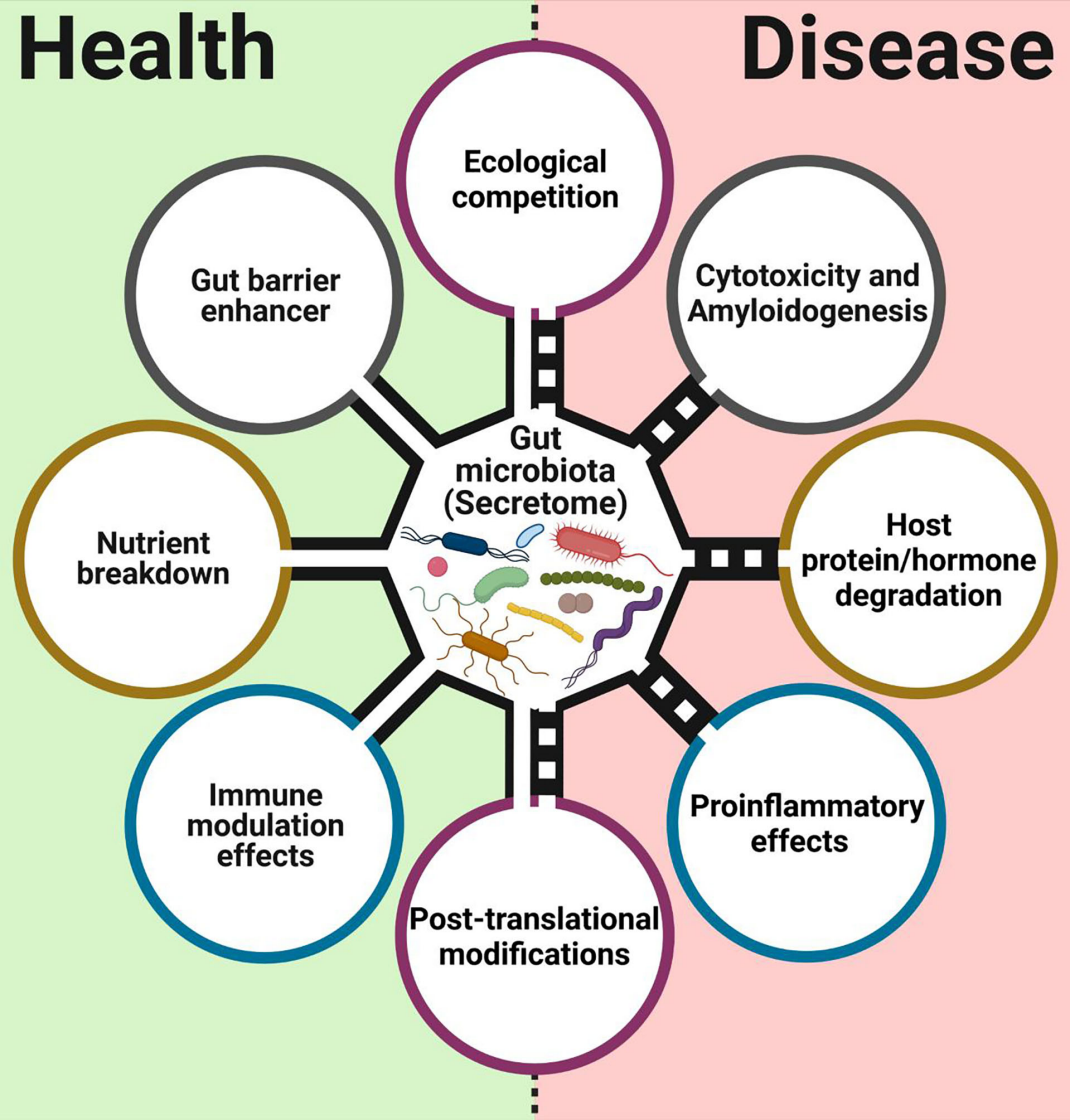
Main functions associated with proteins, enzymes, and surface layer proteins from commensals and probiotics bacterial intestinal. This figure was created with BioRender.com.

There is an increasing number of reports about the presence and function of different microbial secreted proteins and enzymes in the mammalian gut, with implications for health and disease (**Figure 1**). The first functions of secreted proteins observed in gut commensals were enzymatic. Gut commensals are constantly secreting different proteins to their niche to break different compounds. Among them, secreted carbohydrate-active enzymes (CAEs) are important proteins utilized by gut microbiota to degrade carbohydrates that the host cannot consume (El Kaoutari et al., 2013). Gut microbiota can also produce secreted protease activities influencing the activity of the host, as seen in early studies with a set of *Bacteroides* strains, which could reduce maltase and sucrase activities from human brush-border preparations by degradation (Riepe et al., 1980). This secreted bacterial protease activity could be involved in the brush border damage observed during bacterial overgrowth syndromes. More recently, meta-omic studies showed a functional connection between this secretable protease activity and the severity of ulcerative colitis (UC) (Mills et al., 2022).

Despite being more researched in pathogens, some commensals were also analyzed in their capability to create adhesion structures from their secreted proteins. *Veilonella parvula*, a potential roleplayer in developing infant gut immunity, can secrete an autotransporter to auto-aggregate during biofilm formation to compete with other members of the microbial community (Béchon et al., 2020).

Some secreted proteins can produce posttranslational modifications to host proteins. O-linked N-acetylglucosaminylation (O-GlcNAcylation) by either the O-GlcNAcase (OGA) or the O-GlcNAc transferase (OGT) enzymes is predicted as a widely distributed feature in among members of Bacteroidetes and Firmicutes phyla, and depleted in the metagenome of subjects with UC (He et al., 2021). Moreover, microbial OGAs can influence the NF-κB signaling pathway, as observed in *in-vitro* studies, suggesting that those microbial enzymes can also influence the host (He et al., 2021). Some proteins from the microbiome can degrade peptide hormones: another *in-vitro* study showed that a protein called GelE, isolated from *Enterococcus faecalis*, is able to degrade the glucagon-like peptide-1 (GLP-1), a gastrointestinal hormone responsible for the regulation of appetite and glucose homeostasis (LeValley et al., 2020). This evidence points to a higher perspective about how microorganisms can interact directly with host proteins.

Another role observed from secreted proteins is their function as regulators of microbial communities. It has been observed that some organisms can induce inter- or intra-species antagonisms *via* the effect of some secreted proteins, like in the well-known case of the bacteriocins, peptides isolated from several gut commensals, a matter that has been addressed in other reviews (Dicks et al., 2018; Garcia-Gutierrez et al., 2019); several probiotics also contain bacteriocins, which some authors consider this as a probiotic trait (Deliorman Orhan, 2021). Some authors have even hypothesized that bacteriocins may pass the gut–blood barrier (Dicks et al., 2018), a feature with important implications for human infectious diseases. In the case of the commensals, strains from different gut organisms such as *Enterococcus faecium, E. faecalis*, *Escherichia coli*, *Lactobacillus salivarius*, *L. acidophilus*, *L. gasseri*, *L. johnsonii*, *L. reuteri*, and *L. rhamnosus* were found to produce different bacteriocins, with verified antimicrobial activity over members of the same or different species (Garcia-Gutierrez et al., 2019). In members of Bacteroidales, the role of secreted antimicrobial proteins (called BSAPs) has been observed even from genomic, experimental, and metagenomic data, suggesting that those proteins are toxic to members of the same species, mediating strain-level competition (Roelofs et al., 2016). Additionally, in *B. fragilis* strain 638R, the secretion of a highly expressed eukaryotic-like ubiquitin protein (named BfUbb), could inhibit growth from other related strains, suggesting that this protein is also a competition factor with an influence on the community composition (Chatzidaki-Livanis et al., 2017).

Secreted proteins are also associated with pathobionts. For example, in the case of the potential link between bacterial toxins and neurodegenerative diseases (Zhao et al., 2017; Zhao et al., 2021), some strains of *B. fragilis* secrete some proteins such as proteolipids, endo- and exotoxins, and fragilysin, a zinc-metalloproteinase with high neurotoxic activity. This latter toxin is also connected with potential effects in cancer (Haghi et al., 2019) and even with epigenetic changes (Allen et al., 2019). Other secreted proteins may contain amyloid-like properties, with implications for cancer and neurodegenerative disorders (Zhao et al., 2017), among other diseases. For example, FadA, secreted by *Fusobacterium nucleatum*, was associated with microbe reprogramming from commensal to pathogen phenotype during colorectal cancer development (Huang et al., 2022). Moreover, other amyloid-like proteins produced by some gut microorganisms may promote the formation of α-synuclein amyloids, a key etiologic factor for Parkinson’s disease (Wittung-Stafshede, 2022). One reported case of these amyloidogenic proteins is the curli protein CsgA from *E. coli* strain MC4100 (Sampson et al., 2020). A protein of *Bacillus subtilis*, called subtilisin, can translocate the gut epithelial barrier and cleave the human transthyretin (hTTR), generating a fragmented version with amyloidogenic properties with a potential role in senile systemic amyloidosis (SSA), a known factor involved in cardiovascular diseases (Peterle et al., 2020). Moreover, the protein fraction of the conditioned medium (i.e., the cell-free medium containing biologically active components derived from previous cell growth) from the potential probiotic *Akkermansia muciniphila*, cultivated in mucin-depleted conditions, could induce a set of phenotypic changes that could lead to alpha-synuclein aggregation, a pivotal feature associated Parkinson’s Disease development (Amorim Neto et al., 2022). This result raises the question of how potentially probiotic members of the gut microbiota may generate negative outcomes due to the effect of their secretable proteins.

Finally, as previously mentioned in the case of bacteriocins, there are discovered several secreted proteins found in probiotic microorganisms. Probiotics are defined as “live microorganisms which, when administered in adequate amounts, confer a health benefit on the host” (Hill et al., 2014; Reid, 2016). Probiotics have an extended repertoire of functions supporting their role in host health, and the secretion of bioactive proteins is one of those probiotic properties. A repertoire of proteins and enzymes secreted from some strains of commensals and probiotics has been discovered and studied (**Table 1**), and the compilation of their functions in the human gut has been previously reviewed (Sánchez et al., 2010). The main functions associated with the secreted proteins are the protection of the gut barrier (with concomitant reduction of barrier permeability), nutrient degradation (via catabolism of dietary polysaccharides), immunomodulation (especially in an anti-inflammatory manner), cell growth arrest, stimulation of hormones involved in metabolism regulation and competition against pathogens, among other roles (**Table 1**). It is worth to notice that several species contain characterized proteins with the reported effect produced by their probiotic sources (**Table 1**). A remarkable example is *Akkermansia muciniphila*, with the proteins Amuc_1100 and P9, with immunoregulatory and metabolic-regulating properties, aspects already reported for the role of this organism in murine models and human subjects (Everard et al., 2013; Depommier et al., 2019). It is also worth to indicate that microbial protein secretion also can be induced by host cells: a previous *in-vitro* study showed that the presence of colonic epithelial cell-derived vesicles could induce *Lactobacillus rhamnosus* GG, a widely studied probiotic strain, to secrete a protein named P40, a protecting agent of intestinal epithelial cells (Yang et al., 2019). This observation suggests a strong connection between some probiotic microbes (formerly isolated from human samples), and human cellular products.

**Table 1:** Studied proteins and enzymes found in commensals and probiotics bacterial intestinal.

Protein/Enzyme	Bacteria (strain)	Role/Effect	Reference
Amuc_1100	*Akkermansia muciniphila* (ATTC BAA-835^T^)	TLR2 activation and increased *cldn3* and *ocln* expression/Gut barrier integrity	(Plovier et al., 2017)
P9	*Akkermansia muciniphila* (ATTC BAA-835^T^ and SNUG-61027)	Binding to ICAM receptor, increased thermogenesis and stimulation of GLP-1 secretion/Involved in the metabolic control of the host	(Yoon et al., 2021)
OGA	*Akkermansia muciniphila* (EB-AMDK-3) and *Bacteroides thetaiotaomicron* (KLE1254)	Hydrolys O-GlcNAcylated NF-κB-p65 subunit/Regulates proinflammatory processes	(He et al., 2021)
BfUbb	*Bacteroides fragilis* (638R)	Mediates intraspecies antagonism	(Chatzidaki-Livanis et al., 2017)
BSAP-2	*Bacteroides uniformis* (CL03T00C23)	Inhibition of intestinal colonization in members of the same species of different strains	(Roelofs et al., 2016)
B7	*Bifidobacterium longum* subsp. *longum* (ATCC 15707^T^)	Reduces CCR2 expression/Inflammatory environment prevention	(Fernández-Tomé et al., 2019)
020402_LYZ M1	*Bifidobacterium longum* (020402)	Modulates the composition of human gut microbiota	(Du et al., 2021)
Enterocin A+B	*Enterococcus faecium* (por1)	Antibacterial activity, degradation of biofilm formation, inhibition of cancer cell growth	(Ankaiah et al., 2017; Ankaiah et al., 2018)
SagA	*Enterococcus faecium* (Com15)	Endopeptidase that generates small muropeptides that activate NOD2/Improves intestinal barrier function, host immunity, and promotes tolerance to pathogens	(Rangan et al., 2016; Kim et al., 2019)
DegP	*Escherichia coli* (Nissle 1917)	Protease and chaperone/Controls the formation of biofilms	(Fang et al., 2018)
TcpC	*Escherichia coli* (Nissle 1917)	Increasing transepithelial resistance, upregulation of Cldn14 *via* PKCζ and ERK1/2 signaling pathways/Improves intestinal barrier function	(Hering et al., 2014)
MAM	*Faecalibacterium prausnitzii* (A2-165)	Inhibition of the NF-κB pathway/Regulates proinflammatory processes	(Quévrain et al., 2016)
MIMP	*Lactobacillus plantarum*	Decreasing of *IFN-γ*, *IL-*17 and *IL-*23, and increasing of *IL-*4, *IL-*10, Ocln, Zo1 and Jam1/Inhibition of intestinal inflammation and restoration of intestinal lesions	(Yin et al., 2018)
STp	*Lactobacillus plantarum* (BMCM12)	Decreased IFNγ and increased IL-10/Immune response/tolerance mechanisms in the gut	(Bernardo et al., 2012)
HM0539	*Lactobacillus rhamnosus* (GG)	Upregulation of Zo1, Ocln and Muc2, and permeability reduced/Protection of intestinal function	(Gao et al., 2019)
Llp1 and Llp2	*Lactobacillus rhamnosus* (GG)	Inhibition of biofilm formation in different bacterial pathogens	(Petrova et al., 2016)
P40 and P75	*Lactobacillus rhamnosus* (GG)	Activates the PKB/Akt pathway/Prevent apoptosis	(Yan et al., 2007)
P8	*Lactobacillus rhamnosus* (KCTC 12202BP)	Inhibition of Cdk1/Cyclin B1 activation *via* the p53-p21 pathway/Cell growth arrest	(An et al., 2019)
Hld	*Staphylococcus epidermidis* (JA1)	Stimulation of GLP-1 release through a calcium-dependent mechanism/Reduction of markers of obesity and Type 2 Diabetes Mellitus	(Tomaro-Duchesneau et al., 2020)
*β*-galactosidase	*Streptococcus thermophilus* (ATCC 19258^T^)	Reduction of tumor formation, decrease of cell proliferation and mediates inhibition of the Hippo pathway/Tumor suppressor	(Li et al., 2021)

Furthermore, some experiments additionally suggest that there are actually several unidentified secretable proteins involved in the probiotic effect of some commonly used agents (Sánchez et al., 2010). Those unidentified mixtures showed improving effects on gut barrier integrity (Ewaschuk et al., 2008), or amelioration of enteropathogenic infection (Broekaert et al., 2007), among other effects.

## Secretable proteins as a source of postbiotics

The use of the term “postbiotic” has increased in recent years (Wegh et al., 2019). A postbiotic agent can be defined as a “preparation of inanimate microorganisms and, or, their components conferring a health benefit on the host” (Salminen et al., 2021). Secreted proteins enter the scope of this concept. Some studies have already made connections between the role of some beneficial secreted proteins and their potential use as postbiotics, such as the case of the protein HM0539 from *Lactobacillus rhamnosus* GG (Gao et al., 2019). Postbiotics can be discovered by evaluating the effect of dead probiotic cells on the host. For example, the role of the Amuc_1100 protein (**Table 1**) was investigated after it was observed that pasteurized extracts from *A. muciniphila* could produce even a higher effect than the live organism (Plovier et al., 2017). The use of specific secretable proteins as postbiotics may exhibit some advantages compared with the use of probiotics (Nataraj et al., 2020). For example, probiotics involve the use of living and independent organisms that must be controlled and directed to specific objectives, often in a strain-dependent manner. Due to safety reasons (Sanders et al., 2010), the use of genetically-modified probiotics in human subjects is subject to controversies (Plavec and Berlec, 2020). Postbiotic agents can overcome this limitation: as well as several secretable proteins can be purified, and several of them exhibit a similar effect directly; in comparison with the live microbial source, the use of the purified secreted protein can offer new standards for safety, with the same beneficial effects for human health. Moreover, the production of isolated proteins as postbiotics also involves easier storage and generation strategies than the storage and production of living organisms, with concomitant effects on the scalability of mass production at an industrial level.

## Conclusions and horizons on secreted proteins research

The evidence presented in this minireview shows that secreted proteins produced by the gut microbiota may influence the host in positive and negative manners. In the first case, the discovery of new secreted proteins utilized by pathobionts can help to find new therapeutic targets to attack during certain diseases. On the other hand, the discovery of beneficial proteins secreted by commensals or probiotics may lead to the development of novel postbiotic products.

Today, there are several methods available to discover new secreted proteins. In addition to the classic “conditioned medium” experiments, utilized in the past to discover single proteins or undefined mixtures (Sánchez et al., 2010), the arrival of comparative genomics, transcriptomics, and metagenomics have become powerful allies in the discovery and characterization of more secreted proteins. For example, *in-silico* reconstruction of the secretome has been made from the pangenome of *Lactobacillus rhamnosus* (Kant et al., 2014), or for the *Bifidobacterium* genus (Lugli et al., 2018), relevant groups of the human gut microbiota organisms including probiotic agents, formulating the concept of the *pansecretome*. This type of reconstruction can also be made from metagenomes or even metatranscriptomes. For example, a study using a set of obese and normal-weight Mexican children metatranscriptomes detected the set of potentially secreted proteins using an *in-silico* approach from the content of those RNA sequenced samples (Gallardo-Becerra et al., 2020). This latter study formulated the concept of *secrebiome*.

By the use of transcriptome analyses, the expression pattern of some gut microbiota members can be also directed to the search for valuable secretable protein production patterns. A transcriptomic study comparing *Akkermansia muciniphila* expression in the presence or absence of mucin (Shin et al., 2019) showed the upregulation of 79 genes encoding secreted protein candidates during mucin depletion (including the postbiotic agent Amuc_1100). Those transcriptome changes were consistent with the previous observation that *A. muciniphila* grown under mucin-depleted conditions is capable of reducing obesity and improving intestinal barrier integrity in high-fat diet-induced diabetic mice.

Furthermore, metagenomics is also useful for discovering previously unknown genes with the potential to be secreted proteins. A massive (1,773 samples) metagenomic screening searching for novel small genes (Sberro et al., 2019) showed that near 30% of a set of ~4,000 novel gene families were predicted to have a signal peptide or have transmembrane regions, suggesting that a considerable proportion of novel gene families in the human microbiome could be targeted to be secreted.

Finally, by either using classical biochemical/molecular techniques or by combining those strategies with “-omic” disciplines, the ability to discover new secreted proteins opens the possibility of more functional secreted proteins that may influence the host.

## Author contributions

JC conceived the study; BV-V, DG, and JC gathered the data. BV-V made the table and the figure. JC wrote the paper. All the authors read the manuscript and approved the content.

## Funding

JC research is supported by Fondecyt ANID project #11200209. BV-V is supported by ANID Grant For Doctorate Studies in Chile (*ANID Doctorado Nacional 2021-21211564*).

## Conflict of interest

The authors declare that the research was conducted in the absence of any commercial or financial relationships that could be construed as a potential conflict of interest.

## Publisher’s note

All claims expressed in this article are solely those of the authors and do not necessarily represent those of their affiliated organizations, or those of the publisher, the editors and the reviewers. Any product that may be evaluated in this article, or claim that may be made by its manufacturer, is not guaranteed or endorsed by the publisher.
